# Acknowledgement for Reviewing

**DOI:** 10.14252/foodsafetyfscj.D-21-00027

**Published:** 2021-12-24

**Authors:** Food Safety Commission of Japan

The Editor-in-Chief expresses his gratitude to the following individuals, who reviewed manuscripts for Food Safety Vol. 7 to Vol. 9.

